# Clinique of Atlanta Medical College

**Published:** 1871-05

**Authors:** John O. Perkins

**Affiliations:** Dispensary Clerk


*Clinique of Atlanta Medical College.* Service of Dr. J. G. WESTMORELAND.

[Reported by John O. Perkins, Dispensary Clerk.]

---

*Case* 1.—A colored woman, *æt* twenty-five, has never borne children, and is apparently stout. Diagnosis: *Chronic Endo-Metritis.*

The case before you, gentlemen, has been under treatment two months, irregularly. Only local means have been used, and a decided improvement has been made. The almost complete relief she makes known, from pain in the back, soreness in lower portion of the abdomen, and the excessive leucorrhœal discharge, of which at first she complained, shows decided abatement of the intra-uterine chronic inflammation. The very slight discharge of transparent mucus from the os uteri, as exhibited in the speculum, shows great improvement since our first examination, two months since. The test just made by the application of solution of nitrate silver, producing opacity and the leaden hue of the discharge, proves, however, that disease still exists in the internal uterine mucous membrane. The catheretic which I propose now to apply to this surface, and which has been used three times previously, is composed of Lugol's solution of iodine five parts, glycerine three parts, and two grains of acetate morphia to a fluid ounce of the mixture. The hard rubber graduated uterine syringe I present to you, as at present graduated, contains, when charged, about sixteen drops of this preparation. The nozzle will now be inserted to the fundus, and, on withdrawing it, three or four drops discharged at points in the cavity from half inch to one inch apart. This manner of injecting diffuses more generally over the surface the injection used, so that the whole membrane may be impressed, and with less danger of producing uterine colic than the sudden discharge of all at one point. Observation proves that this mixture of iodine does not so readily unite with the mucus as a solution of nit. arg., and therefore more readily comes in contact with the mucous membrane. On this account the remedy selected to-day is, perhaps, more liable to give pain when inflammation exists.

This case, at most, will not, perhaps, require more than one other application of the remedy to relieve completely the uterine disease. When this is accomplished, we have reason to expect relief from the train of nervous symptoms which attend such local disease. In a measure they have already subsided, according to the patient's report.

*Case* 2.—A girl, fourteen years old—somewhat emaciated, and complains of distressing cough, particularly at night.

By inspection, it is ascertained that the left side of the chest, just below the clavicle, is depressed. Percussion gives dull sound, and anscultation, bronchial respiration at this point. The history, as given by the mother, dates the commencement of pulmonary disturbance some two months since, introduced by symptoms of ordinary "bad cold." The cause operating in this case is not certainly known; but although commencing with symptoms of catarrh, the location of disturbance being in the apex of the lung, tuberculous origin may reasonably be suspected. Chronic pneumonia, from the gradual extension of inflammation along the bronchia into the parenchymatous structure is possible, but not very probable.

In the adoption of treatment in this case, we have three objects in view. Firstly, to allay the chronic inflammation in the lung; secondly, to remove the deposit which may exist in the lung, and similar matter in the blood or elsewhere, by catalysis; and thirdly, to prevent the liability to further generation of such substance. The first two must be effected by catheretic and catalytic means. The latter by general invigoration of the system to a point of health and strength unfavorable to the further production of tuberculous matter, by nourishing diet and stimulants of the nervous system. In iodine we have both catheretic action on local inflammation and catalytic influence upon tuberculous substance. By the inhalation of this remedy, not only may the local effect upon the diseased portion of lung be realized, but, through the absorption which necessarily takes place to some extent in the respiratory organs, the general, alterative, or, more properly speaking, catalytic influence, may be had. Being equal in these respects to any other agent, and much more readily introduced into the lungs than other remedies of the kind, the prescription in this case will be the regular daily use of iodine by inhalation. Simple means are sufficient to carry out this process. A large-mouthed, short, one or two-ounce vial is equal, for this purpose, to the most expensive and complicated apparatus. We will now use an ordinary drachm morphine bottle, which, though rather small, will answer to instruct the mother in the manner of using it at home. A scale or two of iodine placed in the vial will, when held over the lamp, 'cause sufficient fumes for one dose. By closing one nostril and inhaling through the other forcibly, and alternating two or three times, the process will be concluded and a sufficient amount taken for one time.

*Case* 3.—A colored girl, *æt* about twenty years—complains of pain in the back and hips; has slight leucorrhœal discharge. Menstruation has been suppressed three months.

In this case, gentlemen, we have a representative of a class which always proves embarrassing to the young practitioner. Uterine disease sometimes, though rarely, causes complete suppression of the catamenia. Pregnancy arrests this function, and one of the most reliable evidences of this state, previously to the fourth month, is the absence of the regular menstrual discharge. The opinion prevails among the people generally that remedies calculated to promote menstruation, when suppressed from disease, will also produce abortion, and wicked females sometimes feign disease with the evil purpose of obtaining emmenagogue prescriptions, hoping thereby to destroy their innocent offspring. Now, while some of the means used to correct disturbed menstruation will not in the least affect the gravid uterus, others, which powerfully excite the pelvic viscera, may lead to premature contraction and expulsion of its contents.

In the case before us, there is probably no evil design in asking treatment—nevertheless, the adoption of a course calculated to disturb an impregnated womb, would show inexcusable carelessness on my part. Hence the expectant plan adopted, viz: mild astringent vaginal wash, and the injunction that she return to the Dispensary occasionally till with certainty her true condition may be known by the period of quickening or otherwise.

Such cases sometimes present themselves in unmarried females whose chastity has not been questioned; and while any expression, by the physician, indicating a doubt of her innocence would be highly reprehensible, yet the use of uterine stimulants, or other unnecessary exciting means, is equally improper.

